# *Persea americana* extract protects intestinal tissue from *Eimeria papillata*-induced murine Infection

**DOI:** 10.1186/s12917-023-03810-1

**Published:** 2023-11-28

**Authors:** Fatemah Alajmi, Tahani Al-Otaibi, Saleh Al-Quraishy, Esam M. Al-Shaebi, Nawal Al-Hoshani, Mohamed A. Dkhil, Rewaida Abdel-Gaber

**Affiliations:** 1 Department of Biology, College of Science, University of Hafr Al Batin, Hafr Al Batin, P.O. 39524, Saudi Arabia; 2 Department of Science and Technology, Al-Nairiyah University College, University of Hafr Al-Batin, Hafr Al- Batin, 31991 Saudi Arabia; 3 Department of Zoology, College of Science, King Saud University, P.O. 2455, Riyadh, 11451 Saudi Arabia; 4 Department of Biology, College of Science, Princess Nourah bint Abdulrahman University, P.O. Box 84428, Riyadh, 11671 Saudi Arabia; 5 Department of Zoology and Entomology, Faculty of Science, Helwan University, Cairo, Egypt

**Keywords:** Mice, Coccidia, Oxidative status, Apoptosis, Natural sources

## Abstract

Coccidiosis is the most prevalent disease-causing widespread economic loss among farm and domestic animals. Currently, several drugs are available for the control of this disease but resistance has been confirmed for all of them. There is an urgent need, therefore, for the identification of new sources as alternative treatments to control coccidiosis. The present work aimed to study the effect of the *Persea americana* extract (PAE) as an anti-coccidial, anti-oxidant, and anti-apoptotic modulator during murine intestinal *Eimeria papillata* infection. A total of 25 male mice were divided into five groups, as follows: *Group1*: Non-infected-non-treated (negative control), *Group2*: Non-infected-treated group with PAE (500 mg/kg b.w). *Group3*: Infected-non-treated (positive control), *Group4*: Infected-treated group with PAE (500 mg/kg b.w.), and *Group5*: Infected-treated group with Amprolium (120 mg/kg b.w.). Groups (3–5) were orally inoculated with 1 × 10^3^ sporulated *E. papillata* oocysts. After 60 min of infection, groups (4 and 5) were treated for 5 consecutive days with the recommended doses of PAE and amprolium. The fact that PAE has an anti-coccidial efficacy against intestinal *E. papillata* infection in mice has been clarified by the reduction of fecal oocyst output on the 5^th^ day post-infection by about 85.41%. Moreover, there is a significant reduction in the size of each parasite stage in the jejunal tissues of the infected-treated group with PAE. PAE counteracted the *E. papillata*-induced loss of glutathione peroxidase (GPx), superoxide dismutase (SOD), and total antioxidant capacity (TCA). *E. papillata* infection also induced an increase in the apoptotic cells expressed by caspase-3 which modulated after PAE treatment. Moreover, the mRNA expression of the goblet cell response gene, mucin (MUC2), was upregulated from 0.50 to 1.20-fold after treatment with PAE. Based on our results, PAE is a promising medicinal plant with anti-coccidial, anti-oxidant, and anti-apoptotic activities and could be used as a food additive.

## Introduction

Intestinal coccidiosis is a cosmopolitan disease affecting a wide variety of vertebrates [1, 2]. The causative agent for this protozoan disease is the apicomplexan species within the genus *Eimeria* (family Eimeriidae). Ernst et al. [3] identified *Eimeria papillata* as a coccidian parasite in the house mouse *Mus musculus*. Infection with this protozoan parasite occurs via the fecal-oral route of oocysts with a high degree of host specificity [4]. The most commonly used method for the detection of coccidia is the flotation technique of oocysts shed in feces [5]. This parasite species, *E. papillata*, spends its life cycle within the intestinal tract causing extensive damage to the intestinal mucosa, inflammation, and oxidative stress that affects general body performance [6, 7].

Therapeutic tools for coccidiosis have relied on the availability of more than 30 anticoccidial drugs [8, 9]. However, the intensive use of these drugs has led to side effects on animal health and the development of drug-resistant *Eimeria* strains [10]. Researchers’ efforts are now directed toward finding alternative agents with no side effects on the host infected with *Eimeria* species [11]. Among, other available options, different compounds obtained from botanicals have shown excellent and admirable anticoccidial and other therapeutic effects [12]. In Saudi Arabia, natural sources such as *Allium sativum* [13, 14], *Phoenix dactylifera* [15], *Punica granatum* [16], *Ziziphus spina-christi* [17], *Salvadora persica* [18–20], *Morus nigra* [21], *Zingiber officinale* [22, 23], and *Azadirachta indica* [24–26] have been evaluated as alternative controls to murine coccidiosis.

*Persea americana*, also known as aguacate (avocado), belongs to the family Lauraceae. The fruits are edible, while the bark, leaves, stem, and roots are utilized as a local remedy [27]. Phytochemical analysis of avocados has revealed a variety of bioactive compounds including phenolics, flavonoids, carotenoids, tannins, saponins, alkaloids, vitamin C, and vitamin E [28, 29]. The medicinal properties attributed to *P. americana* include anti-hypertensive [30], hepatoprotective [31], anti-ulcer [32], anti-cancer [33], insecticidal [34, 35], anti-microbial [36–40], anti-oxidant [41], antidiabetic [42, 43], anti-inflammatory [44–46], and anti-coccidial properties [47].

In this study, the role of *P. americana* extract was investigated against the expression of the cysteine aspartic acid protease-3 (caspase-3), the goblet cells regulating gene, and the oxidative damage caused by *E. papillata* infection in mouse jejunum.

## Materials and methods

### Preparation of the avocado peel extract

*Persea americana* (avocado) fruits were purchased from the local markets in Riyadh, Saudi Arabia. Edible pulps were removed, cut into pieces, air-dried at 40ºC, and then pulverized using an electrical grinder. The obtained powder (100 g) was macerated using 1000 ml methanol (70%) for 42 h with vigorous shaking. The methanolic *P. americana* extract (PAE) was filtered and evaporated under reduced pressure [16]. PAE was dissolved in distilled H_2_O to be used for experimental steps.

### Determination of phenolic and flavonoid contents

The total phenolic content was determined using the Folin–Ciocalteu technique as described by Abdel Moneim [48]. Absorbance was measured at 760 nm with a spectrophotometer (PD 303 UV spectrophotometer, Apel Co., Limited, Saitama, Japan). The measured value was compared to a calibration curve built with gallic acid solutions, and the results are given as mg gallic acid per gram of dry extract (mg GAE/g). Moreover, the total flavonoid content was determined using the aluminum chloride colorimetric method of Abdel Moneim [48]. Absorbance at 510 nm was measured. The flavonoid value was calculated using a calibration curve and reported as mg quercetin per gram dry extract (mg QE/g).

### The 2,2-Diphenyl-1-picrylhydrazyl (DPPH) radical scavenging activity

The activity of PAE was determined to scavenge DPPH radicals according to Akillioglu and Karakaya [49]. Absorbance was measured at 515 nm using a microplate reader (ELX 800; Bio-Tek Instruments, Winooski, VT, USA). The antioxidant activity is expressed as suppression % of DPPH radicals.

### Passaging of *Eimeria* species

*Eimeria papillata* was used as a model murine coccidian parasite and obtained from Prof Heinz Mehlhorn (Heinrich-Heine-Universitat, Germany). Five laboratory mice (*Mus musculus*) were obtained from the animal house at the Department of Zoology (College of Science, King Saud University) and inoculated with 1 × 10^3^ sporulated *E. papillata* oocysts by oral gavage. On the 5th day post-infection (p.i.), feces were collected and sporulated in 2.5% (*w/v*) potassium dichromate (K_2_Cr_2_O_7_) at room temperature [50]. The sporulated oocysts were washed in a phosphate buffer solution (PBS) and used in this experiment. Using an Olympus B×61 microscope (Tokyo, Japan), oocysts (sporulated and non-sporulated) were photographed and described using the guidelines of Duszynski and Wilber [51].

### Experimental design

Twenty-five male C57BL/6 mice (10–12 weeks) were obtained from the College of Pharmacy at King Saud University. All mice have been bred under specified pathogen-free conditions and fed a standard diet and water *ad libitum*. Mice were divided into five groups (5 mice/group), as follows: *Group1*: Non-infected-non-treated (negative control), *Group2*: Non-infected-treated group with PAE (500 mg/kg b.w). *Group3*: Infected-non-treated (positive control), *Group4*: Infected-treated group with PAE (500 mg/kg b.w.), and *Group5*: Infected-treated group with Amprolium (120 mg/kg b.w.). Groups (3–5) were orally inoculated with 1 × 10^3^ sporulated *E. papillata* oocysts in 100 µl of physiological saline. After 60 min of infection, groups (4 and 5) were treated for 5 consecutive days with the recommended doses of PAE and amprolium via oral gavage in 100 µl based on the previous study of Al-Otaibi et al. [47].

### Sample collection

On the 5^th^ day p.i., fresh fecal pellets of each mouse from all experimental groups were collected separately and examined for the presence of *E. papillata* oocysts. According to Schito et al. [52], the number of oocysts per gram of feces was estimated using the McMaster technique. Additionally, the suppression (%) of oocyst shedding was calculated as follows: 100 – (oocysts output in the treated group/oocysts output in the infected group) × 100.

### Histological examination of parasite stages

On the 5th day p.i., CO_2_ asphyxia was used for the euthanasia of all experimental animals. To evaluate the morphometric changes among the *Eimeria* stages in the mouse jejunum, pieces of jejuna were collected after dissection on the 5^th^ -day p.i. of mice and fixed in formalin (10%) for 24 h, dehydrated and embedded in paraffin wax. Sections were cut and stained with hematoxylin and eosin (H&E) [53]. Under an Olympus B×61 microscope (Tokyo, Japan), parasite stages (gamonts and developing oocysts) were observed in sections of the infected and infected-treated groups and then measured using a calibrated ocular micrometer.

### Oxidative status in the jejunum

Parts of jejunumwere weighed and homogenized in an ice-cold medium of 50 mM Tris-HCl and 300 mM sucrose. The mixture was centrifuged for 10 min (500×g and 4 ºC) to give a final yield of 10% (w/v) jejunal homogenate and then kept at -20 °C until use [54]. For different biochemical assays, the supernatant was used and evaluated colorimetrically to determine glutathione peroxidase (GPx) [55], superoxide dismutase (SOD) [56], and total antioxidant capacity (TAC) [57] with its related kits (Biodiagnostic Co., Egypt). The absorbance of the reactions was measured by Molecular Device (Spectra MAX 190) provided with SoftMax® Pro software v. 6.3.1.

### Immunohistochemical staining of *Caspase-3*

Paraffin-embedded jejunal sections were treated with 3% H_2_O_2_ for 10 min, blocked with fetal bovine serum (5%), and then incubated at 4 °C overnight with a primary polyclonal rabbit anti-mouse antibody specific for cysteine aspartic acid protease-3 (*Caspase-3*) (1:100 dilution in PBS, Santa Cruz Biotechnology, CA, USA), according to Dkhil et al. [58]. After triplicate washing with PBS, samples were treated with a biotin-conjugated secondary antibody (1:2,000 dilution in PBS). Sections were counterstained for 1 min with hematoxylin and re-incubated for 15 min with streptavidin which was labeled with horseradish peroxidase. All sections were photographed using an Olympus B×61 microscope (Tokyo, Japan).

### Goblet cell response gene (MUC2) expression

Using Trizol (Invitrogen), total RNA was isolated from the preserved samples (at -80 °C). RNA samples were treated with DNase (Applied Biosystems, Darmstadt, Germany) for at least 1 h and then converted into cDNA using the reverse transcription kit (Qiagen, Hilden, Germany) following the manufacturer’s procedure. Quantitative real-time PCR (qRT-PCR) was performed using the ABI Prism® 7500HT sequence detection system (Applied Biosystems, Darmstadt, Germany) with QuantiTect™ SYBR® green PCR master mix (Qiagen, Hilden, Germany) and the gene-specific primers (Qiagen, Hilden, Germany): goblet cell response gene (*MUC2*) (Mm_Muc2_2_SG, Cat. No. Mm_Muc2_2_SG) and Glyceraldehyde-3-phosphate dehydrogenase (*GAPDH*) (Mm_Gapdh_3_SG, Cat. No. QT01658692). The ^2−ΔΔ^CT method of Livak and Schmittgen [59] was used to evaluate the fold-change in mRNA expression. *GAPDH* was used as a reference gene.

### Statistical analysis

Differences between obtained values (mean ± SD) for experimental groups were compared by two-way analysis of variance (ANOVA) using SigmaPlot® version 11.0 (Systat Software, Inc., Chicago, IL, USA). The *p*-value ≤ 0.05 was considered a statistically significant difference.

## Results

**Fig. 1:**
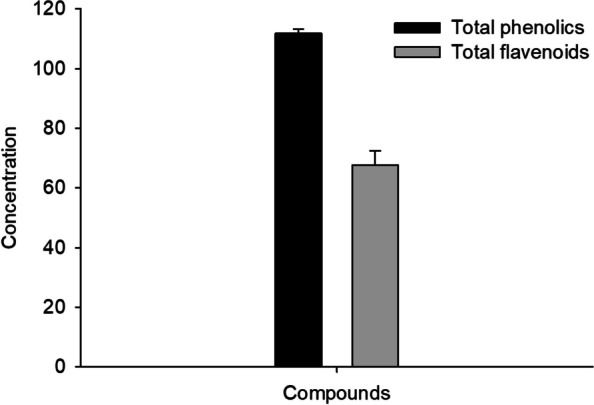
The concentration of phenolics (mg GAE/g) and flavonoids (mg QE/g) in PAE

The total phenolic content in PAE was determined using the Folin–Ciocalteu technique as 111.8 ± 1.38 mg GAE/g (Fig. 1). Moreover, the total flavonoid content in PAE was determined using the aluminum chloride colorimetric method as 67.63 ± 4.85 mg QE/g (Fig. 1). The DPPH radical scavenging activity was 85.53 ± 1.58 for PAE.

**Fig. 2:**
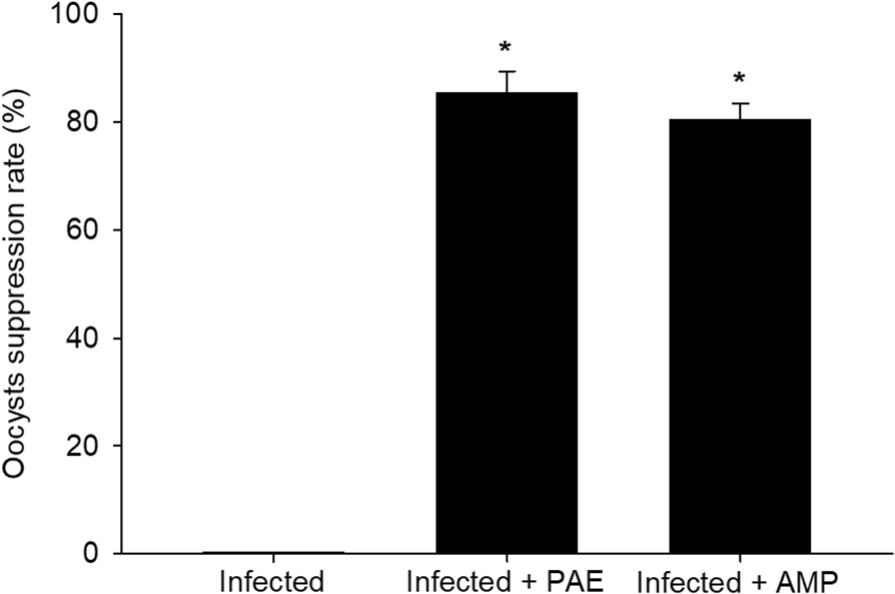
Suppression rate of *E. papillata* oocysts in the infected and infected-treated mice with PAE and AMP groups. Significance at *p* ≤ 0.05 against the infected group (*)

**Fig. 3:**
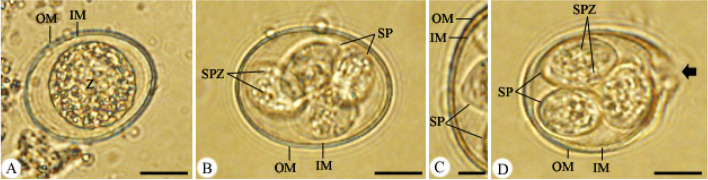
*Eimeria papillata* oocyst **A** non-sporulated oocyst. **B** sporulated oocyst. **C** oocyst bi-layered. **D** site of splitting sporocysts during excystation (black arrow). Scale bar = 10 μm (**A**, **B**, **D**), and 5 μm (**C**), (*OM* Outer membrane, *IM* Inner membrane of oocyst, *Z* Zygote, *SP* Sporocysts, *SPZ* Sporozoites)

Mice in the experimental groups (3–5) started shedding oocysts (non-sporulated) after 3 days p.i. On the 5^th^ day p.i., the *Eimeria* oocyst output was observed to be 4.075 × 10^9^ oocysts/g feces in the infected group, which is associated with general weakness, poor body performance, loss of appetite, and diarrhea. PAE was significantly able to suppress the oocyst output by 85.41% in comparison to 80.38% in the drug-treated group (Fig. 2). Oocysts were sub-spherical and surrounded by a thick bi-layered wall (Fig. 3). After sporulation, four ellipsoidal sporocysts were observed with two sporozoites per each (Fig. 3).

**Fig. 4:**
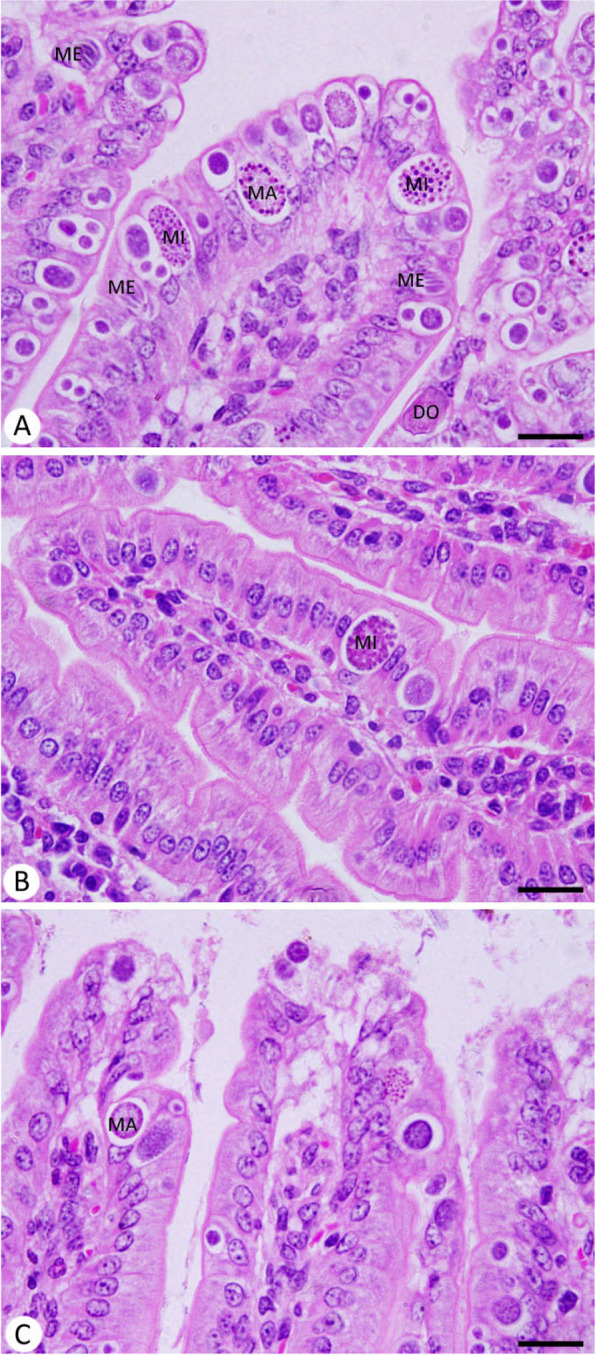
Histology of jejunal tissue of mice in different experimental groups. **A** *E. papillata* infected jejunum with an increased number of parasite stages. **B** and **C** infected treated mouse (PAE and AMP, respectively) with decreased number of parasite stages. *MI* Microgamonts, *MA* Macrogamonts, *ME* Merozoites, *DO* Developing oocyst). Scale bar = 50 μm

The developmental *Eimeria* stages appeared inside the jejunal tissue (Fig. 4). The reduction of oocyst output was due to the impaired development of *Eimeria* stages. In the infected group, microgamonts measured 17.94 ± 0.18 μm, macrogamonts 17.26 ± 0.41 μm, and developing oocysts 18.18 ± 0.13 μm (Table 1). After treatment with PAE, there was a significant morphometrical reduction to 16.67 ± 0.08 μm (microgamonts), 12.87 ± 0.29 μm (macrogamonts), and 15.00 ± 0.19 μm (developing oocysts) in comparison to the drug-treated group.

**Table 1:** Morphometric changes of developmental changes of *Eimeria papillata* in infected and treated groups

Group	Microgamonts	Macrogamonts	Developing oocysts
**Infected group**	17.94 ± 0.18	17.26 ± 0.41	18.18 ± 0.13
**Infected + PAE**	16.67 ± 0.08^ab^	12.87 ± 0.29^ab^	15.00 ± 0.19 ^a^
**Infected + AMP**	16.44 ± 0.09^a^	15.42 ± 0.14^a^	15.48 ± 0.03 ^a^

All values are in micrometers and presented as means ± SD

^a^Significant change concerning the infected group

^b^Significant change concerning the infected + 120 mg/kg AMP group

**Fig. 5:**
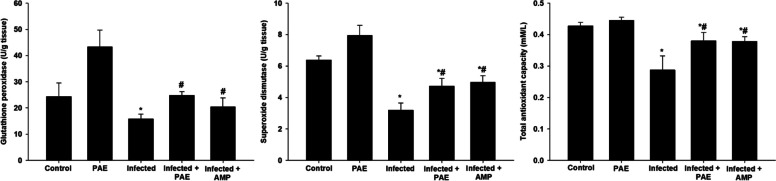
Effect of PAE on glutathione peroxidase, superoxide dismutase, and total antioxidant levels in mouse jejunum infected with *E. papillata*.^*^Significance changes concerning the control group,^#^Significance changes for the infected group

The GPx level significantly declined from 24.31 ± 5.22 in the non-infected group to 15.80 ± 1.79 mg/g tissue in the infected group. While, the level of GPx of mice treated with PAE and reference drug was elevated to 24.80 ± 1.40 and 20.42 ± 3.44 mg/g tissue, respectively (Fig. 5). Moreover, the SOD level significantly declined from 6.38 ± 0.25 in the non-infected group to 3.18 ± 0.46 U/g tissue in the infected group. While, the level of SOD in mice treated with PAE and reference drug was significantly elevated to 4.72 ± 0.48 and 4.95 ± 0.41 U/g tissue, respectively (Fig. 5). There was a significant decline in the level of TAC from 0.42 ± 0.01 in the non-infected group to 0.28 ± 0.04 mM/L in the infected group. While, TAC of mice treated with PAE and reference drug was significantly elevated to 0.37 ± 0.02 and 0.37 ± 0.01 mM/L, respectively (Fig. 5).

**Fig. 6:**
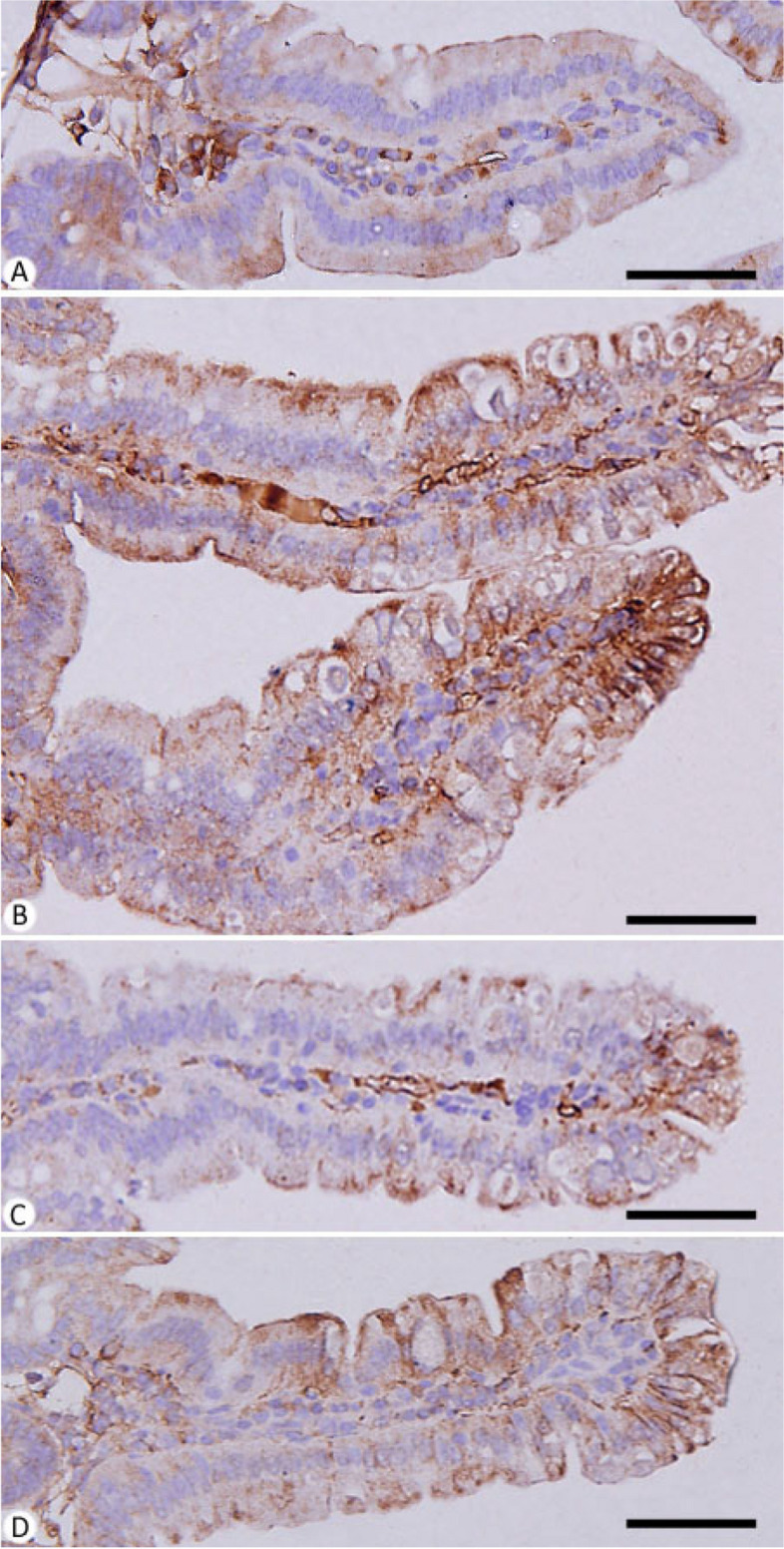
Immunohistochemical localization of caspase-3 in the jejuna of mice. **A** control non-infected jejunum. **B** *E. papillata* infected jejunum with an increased number of caspase-3 positive cells. **C** and **D** infected treated mouse (PAE and AMP, respectively) with decreased number of caspase-3 positive cells. Scale bar = 50 μm

The role of PAE in *Eimeria* infection-induced apoptosis was checked, through the histochemical staining for caspase-3 in the mice jejuna from different experimental groups. Infection with *E. papillata* induced apoptotic changes within the jejunal tissues of the infected mice group (Fig. 6). Immunohistochemical investigation for caspase-3 showed that PAE was able to decrease the immunoreactivity in the jejuna of mice infected with *E. papillata* (Fig. 6).

**Fig. 7:**
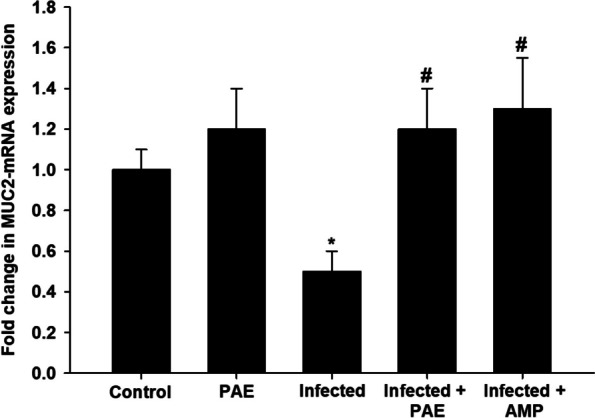
Effect PAE on the mRNA expression of *MUC2* in the jejunal samples from *E. papillata*-infected mice. The expression values obtained by RT-PCR analysis were normalized to the reference gene *GAPDH* mRNA level and are shown as fold induction (in log 2 scale) relative to the mRNA level in the control. ^*^Significance changes concerning the control group, ^#^Significance changes concerning the infected group

qRT-PCR revealed downregulation in the expression level of the *MUC2* gene in the mice jejunum (at 5^th^ -day p.i.) due to *E. papillata* infection (Fig. 7). However, treatment with PAE significantly upregulated the *MUC2* gene expression from 0.50 to 1.20-fold (Fig. 7). Data were normalized to the *GAPDH* mRNA level and shown as fold induction (in log 2 scale) relative to the mRNA level in the control by RT-PCR.

## Discussion

Since ancient times, natural sources including plants have been used in the treatment of various diseases. Avocado is one of the natural sources which have a chemoprotective effect [60–62]. This study showed that PAE is an efficient agent in ameliorating *E. papillata* infection in mice as it could reduce the oocyst shedding rate on 5^th^ -day p.i. by about 85.41%. This inhibition of *Eimeria* infection is known to occur with most anticoccidial drugs. This diminished output suggests that PAE impairs the development of intracellular *Eimeria* stages in the host intestinal cells before the relatively inert oocyst is formed and finally released. The fact that PAE possesses anti-coccidial activity has been previously reported by Al-Otaibi et al. [47] regard to the content of the extract. Ferreira da Vinha et al. [63], and Rahman et al. [29] found that the PAE contains phenolics, flavonoids, carotenoids, tannins, saponins, alkaloids, and vitamins. Significant changes in the size of *Eimeria* stages were observed after PAE treatment. This might be due to the polyphenolic compounds of PAE which exert antimicrobial activity leading to impaired membrane functions and leakage of cellular constituents [64].

Our findings demonstrated *E. papillata* infection is associated with oxidative damage to the mice jejunum, which leads to the depletion of antioxidant enzymes and reduction of GPx, SOD, and TAC which are indispensable for protecting the animal body from the damage caused by free radicals during *Eimeria* infection. Previous studies [14, 15, 63, 65–67] reported that the imbalance of the antioxidant defense system due to *Eimeria* infection leads to harmful cellular effects. The treatment of *E. papillata-*infected mice with PAE significantly resulted in the pronounced modulation of oxidative damage and enhanced antioxidant capacity in the jejunum of mice. These results showed that PAE acts as an excellent antioxidant activity, agreed with Vo et al. [68] stated the presence of free radical scavenging properties in avocados which offer protection against oxidative damage. In our previous study, we proved that glutathione reduced (GSH), nitric oxide (NO), and malondialdehyde (MDA) activities improved in the jejuna of *E. papillata*-infected mice after treatment with PAE due to the presence of phenolic compounds [47].

Previous studies by Lüder et al. [69] and Balamurugan et al. [70] reported that apoptosis could regulate the host response to a variety of intracellular parasitic infections and help to eliminate the infected cells. Alkhudhayri et al. [71] studied the relationship between the developmental stages of *E. papillata* and host apoptosis. In this study, the death of jejunal cells in the infected mice was evidenced by a significant observation of the pro-apoptotic markers of caspase-3. This agreed with Dkhil et al. [24], Metwaly et al. [15], and Abdel-Gaber et al. [25], who reported that parasite invasion and replication may cause considerable stress to the host cells which triggered apoptosis for the infected intestinal cells. Treatment of infected mice with PAE significantly reduced the rate of caspase-3 and improved the apoptotic changes in jejunal cells, this agreed with those stated the anti-apoptotic activity of avocado extracts of Bonilla-Porras et al. [72], Abozaid et al. [73], and El-Magd et al. [74].

Goblet cells (GCs) are considered a dynamic protective agent against pathogens [75]. GCs are produced from stem cells (SCs) that are confined to the intestinal crypts [76]. Inside GCs, the *MUC2* gene is widely expressed and is responsible for the regulation of mucin secretion and inflammatory response in preventing pathogen-induced epithelial injury [18, 24, 77]. Our results of qRT-PCR revealed that the expression of the *MUC2* gene was significantly downregulated in the mice jejuna causing physical contact between *E. papillata* and host cells, which is consistent with previous studies [16, 18, 24, 25, 66, 78, 79]. This result reflects that, during infection, SCs are parasitized and become unable to produce GCs associated with the downregulation of the *MUC2* gene. Previous studies [80–82] reported that the alteration in goblet cells could affect the susceptibility of the *Eimeria*-infected host to limit the capacity of the parasite to penetrate the epithelial cells. PAE, based on our results, was able to alter this downregulation of *MUC2* due to infection. The fact that PAE is effective in ameliorating the upregulation of genes associated with inflammation. This agreed with Al-Otaibi et al. [47] mentioned that PAE has a role in the regulation of goblet cell-producing mucin which helps to improve the inflammatory response to infectious diseases.

## Conclusion

Our data indicate that avocados possess an anti-oxidant and anti-apoptotic activity against murine coccidiosis. PAE could be used with normal animal food as an additive to protect host tissue from injuries induced by various pathogenic infections.

## Data Availability

All the datasets generated or analyzed during this study are included in this published article.
